# Supplementary material for: HIVprotI: an integrated web based platform for prediction and design of HIV proteins inhibitors
Source: J Cheminform. 2018 Mar 9;10:12. doi: 10.1186/s13321-018-0266-y (PMC5845081; doi:10.1186/s13321-018-0266-y)
Supplement: Supplementary file 2 — Additional file 2. Source code of HIVProtI web server. [file 13321_2018_266_MOESM2_ESM.zip › HIVprotI_Source-code/hivprotI/weka-3-6-6/doc/weka/classifiers/mi/supportVector/MIRBFKernel.html]

MIRBFKernel


---


|  |  |  |
| --- | --- | --- |
| **Overview** \| **Package** \| **Class** \| **Tree** \| **Deprecated** \| **Index** \| **Help** \| **Weka's home** | |  |
| **PREV CLASS**   NEXT CLASS | **FRAMES**    **NO FRAMES**     **All Classes** |  |
| SUMMARY: NESTED \| FIELD \| CONSTR \| METHOD | DETAIL: FIELD \| CONSTR \| METHOD |  |


---


## weka.classifiers.mi.supportVector Class MIRBFKernel

```
java.lang.Object
  weka.classifiers.functions.supportVector.Kernel
      weka.classifiers.functions.supportVector.CachedKernel
          weka.classifiers.functions.supportVector.RBFKernel
              weka.classifiers.mi.supportVector.MIRBFKernel
```

**All Implemented Interfaces:**: java.io.Serializable, CapabilitiesHandler, MultiInstanceCapabilitiesHandler, OptionHandler, RevisionHandler

---

``` public class MIRBFKernel extends RBFKernel implements MultiInstanceCapabilitiesHandler ```

The RBF kernel. K(x, y) = e^-(gamma \* <x-y, x-y>^2)


Valid options are:

```
 -D
  Enables debugging output (if available) to be printed.
  (default: off)
```

```
 -no-checks
  Turns off all checks - use with caution!
  (default: checks on)
```

```
 -C <num>
  The size of the cache (a prime number), 0 for full cache and 
  -1 to turn it off.
  (default: 250007)
```

```
 -G <num>
  The Gamma parameter.
  (default: 0.01)
```

**Version:**
:   $Revision: 1.4 $

**Author:**
:   Eibe Frank (eibe@cs.waikato.ac.nz), Shane Legg (shane@intelligenesis.net) (sparse vector code), Stuart Inglis (stuart@reeltwo.com) (sparse vector code), J. Lindgren (jtlindgr{at}cs.helsinki.fi) (RBF kernel), Lin Dong (ld21@cs.waikato.ac.nz) (MIkernel)

**See Also:**: Serialized Form

---

**Constructor Summary**

`MIRBFKernel()`
  
          default constructor - does nothing.

`MIRBFKernel(Instances data,
int cacheSize,
double gamma)`
  
          Constructor.


| **Method Summary** | |
| --- | --- |
| `void` | `buildKernel(Instances data)`             builds the kernel with the given data. |
| `Capabilities` | `getCapabilities()`             Returns the Capabilities of this kernel. |
| `Capabilities` | `getMultiInstanceCapabilities()`             Returns the capabilities of this multi-instance kernel for the relational data. |
| `java.lang.String` | `getRevision()`             Returns the revision string. |

**Methods inherited from class weka.classifiers.functions.supportVector.RBFKernel**

`gammaTipText, getGamma, getOptions, globalInfo, listOptions, setGamma, setOptions, toString`

**Methods inherited from class weka.classifiers.functions.supportVector.CachedKernel**

`cacheSizeTipText, clean, eval, getCacheSize, numCacheHits, numEvals, setCacheSize`

**Methods inherited from class weka.classifiers.functions.supportVector.Kernel**

`checksTurnedOffTipText, debugTipText, forName, getChecksTurnedOff, getDebug, makeCopies, makeCopy, setChecksTurnedOff, setDebug`

**Methods inherited from class java.lang.Object**

`equals, getClass, hashCode, notify, notifyAll, wait, wait, wait`

**Constructor Detail**

### MIRBFKernel

```
public MIRBFKernel()
```

:   default constructor - does nothing.

---


### MIRBFKernel

```
public MIRBFKernel(Instances data,
                   int cacheSize,
                   double gamma)
            throws java.lang.Exception
```

:   Constructor.

    **Parameters:**: `data` - the data to use: `cacheSize` - the size of the cache: `gamma` - the bandwidth **Throws:**: `java.lang.Exception` - if something goes wrong


**Method Detail**

### getCapabilities

```
public Capabilities getCapabilities()
```

:   Returns the Capabilities of this kernel.

    :   **Specified by:**: `getCapabilities` in interface `CapabilitiesHandler` **Overrides:**: `getCapabilities` in class `RBFKernel`
    :   **Returns:**: the capabilities of this object **See Also:**: `Capabilities`

---


### getMultiInstanceCapabilities

```
public Capabilities getMultiInstanceCapabilities()
```

:   Returns the capabilities of this multi-instance kernel for the
    relational data.

    :   **Specified by:**: `getMultiInstanceCapabilities` in interface `MultiInstanceCapabilitiesHandler`
    :   **Returns:**: the capabilities of this object **See Also:**: `Capabilities`

---


### buildKernel

```
public void buildKernel(Instances data)
                 throws java.lang.Exception
```

:   builds the kernel with the given data. Initializes the kernel cache.
    The actual size of the cache in bytes is (64 \* cacheSize).

    :   **Overrides:**: `buildKernel` in class `RBFKernel`
    :   **Parameters:**: `data` - the data to base the kernel on **Throws:**: `java.lang.Exception` - if something goes wrong

---


### getRevision

```
public java.lang.String getRevision()
```

:   Returns the revision string.

    :   **Specified by:**: `getRevision` in interface `RevisionHandler` **Overrides:**: `getRevision` in class `RBFKernel`
    :   **Returns:**: the revision


---


|  |  |  |
| --- | --- | --- |
| **Overview** \| **Package** \| **Class** \| **Tree** \| **Deprecated** \| **Index** \| **Help** \| **Weka's home** | |  |
| **PREV CLASS**   NEXT CLASS | **FRAMES**    **NO FRAMES**     **All Classes** |  |
| SUMMARY: NESTED \| FIELD \| CONSTR \| METHOD | DETAIL: FIELD \| CONSTR \| METHOD |  |


---
